# Force Monitoring in a Maxilla Model and Dentition Using Optical Fiber Bragg Gratings

**DOI:** 10.3390/s120911957

**Published:** 2012-08-29

**Authors:** Maura Scandelari Milczewski, Jean Carlos Cardozo da Silva, Cicero Martelli, Leandro Grabarski, Ilda Abe, Hypolito José Kalinowski

**Affiliations:** 1 Graduate School on Electrical Engineering and Applied Computer Science, Federal University of Technology - Paraná, Curitiba-PR 80230-901, Brazil; E-Mails: mauramil@bol.com.br (M.S.M.); grabarski@gmail.com (L.G.); ildabe.fisica@gmail.com (I.A.); hjkalin@utfpr.edu.br (H.J.K.); 2 Electrical Engineering Graduate Program, Federal University of Technology - Paraná, Pato Branco-PR 85503-390, Brazil; E-Mail: jeanccs@utfpr.edu.br

**Keywords:** orthodontic forces, maxilla, fiber Bragg gratings, optical fiber sensors

## Abstract

The aim of this work is to show the possibility of using fiber optic sensors to instrument inside parts of an artificial maxilla and measure internal tension transmitted by the orthodontic and orthopedic appliances to the dentition and the adjacent bone. Bragg gratings written in a standard optical fiber were used to monitor the maxillary teeth and a multiplexed fiber was used to monitor the surface of the maxillary bone, transversally to the longest axis of the teeth. A Universal Test Machine was used to evaluate the sensitivity of the sensor to the vertical and lateral forces applied on the teeth. A wavelength shift of approximately 0.30 nm was detected when applying loads ranging from 0 to 20 N. By applying forces using the standard orthodontic appliances installed on the dentition it was possible to detect a range of forces between 0.025 N to 0.035 N during the activation of the arch wire and extra-oral forces. The use of the internal sensors in an artificial model made possible the monitoring of the resulting forces on the internal parts of the teeth and at the position where the strain takes place within the maxilla. The sensors detected that the orthodontic forces were not transmitted to the surface of the maxilla. This information is important to elucidate and to correlate undesirable effects as tooth root absorption and local pain during the orthodontic treatment.

## 1. Introduction

The biomechanical process to move teeth to the right positions during orthodontic therapy is to generate pressure on the tooth against the bone, compressing the periodontal ligament (tissue between the bone and the tooth). A suitable compression of the periodontal ligament starts a process of bone remodeling which leads to tooth movement. The pressure is achieved using devices such as brackets and arches with different diameters and designs of coils called orthodontic appliance and extra-oral devices working as an orthopedic appliance. The exact forces applied by the orthodontic and orthopedic systems to the teeth and bone are difficult to determine and remain not well known. One of the most difficult questions in orthodontics is the association between the external applied force and the periodontal ligament response. Understanding the magnitude of the force transmitted to the dentition and bone is important and it is relevant to avoid undesirable effects, such as tooth root absorption, local pain [1], hearing organ pain [2] or pain and discomfort associated with orthodontic anchoring techniques [3].

Optical fiber sensors have been used in different areas of life sciences such as medicine, dentistry and biochemistry (see, e.g., [4–10]). In dentistry, optical fibers and particularly fiber Bragg grating (FBG) sensors have also been used to monitor forces and tensions established by orthodontic appliances throughout the dentition and adjacent bone [11]. Fiber Bragg gratings are suitable to investigate forces in the tooth because of the small dimensions and its sensitivity to transversal loads. To evaluate the forces at several teeth as a function of the load applied to the orthodontic appliances it is possible to use high birefringence (HiBi) optical fiber Bragg gratings [11] or polymeric fiber (PMMA) microbend loss sensors [12] placed between the bracket and the surface of the tooth.

To further improve the information about tooth movement using orthodontic appliances it is important to obtain data about the magnitude of the applied force exactly on the root, this means, the strain that is transmitted from the appliance on the tooth crown to the constitutive sections of the tooth. The present study uses optical fiber Bragg grating sensors instrumented inside of a maxilla model and dentition to analyze such forces. The sensors were embedded into the model during its construction at three principal teeth (according to the level of movement of each one) used for orthodontic treatment. Experimental measurements were firstly done to characterize the system and evaluate the sensitivity of the sensors, and finally to observe the transference of the orthodontic and orthopedic forces through the bone and dentition.

## 2. Maxilla Model and Instrumentation Setup

**Figure 1.**
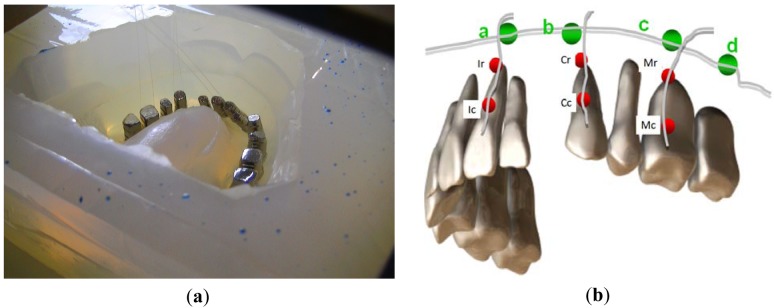
(**a**) A photograph of the mould for maxilla model casting, from center to top left some of the used fibers can be observed as dark lines; (**b**) Schematic drawing of the spatial distribution of the optical fiber Bragg grating sensors at the teeth and maxilla.

To simulate the orthodontic process a maxilla model was constructed using a metal base (typodont) and a set of pre-fabricated metal teeth united by an elastomeric material with well known physical proprieties to try to reproduce the mechanical characteristics of the periodontal ligament and bone. During the construction of the maxilla model [13] the Bragg grating sensors were carefully bonded along the axis of the teeth root, one grating near to the crown and another on the apex (see the red dots in Figure 1(a,b)). Three teeth (the central incisor, canine and molar, all at the left hand side) were instrumented. Another group of four gratings multiplexed in one fiber was placed transversally to the longest axis of the roots on the top of the apex at the surface of the maxilla. The fiber Bragg gratings were labeled arbitrarily according to their positions at the maxilla (Figure 1(b)). The gratings bonded to the roots were identified as Ic, Ir, Cc, Cr, Mc and Mr, consecutively from the incisor root near to the crown (Ic, Cc and Mc) to the molar apex (Ir, Cr and Mr). The gratings a, b, c and d correspond to the multiplexed sensors bonded at the external surface of the elastomeric material (representing the maxilla), transversally to the longest axis of the root near to the apexas shown in Figure 1(b). Table 1 summarizes the sensors positions and the corresponding wavelength.

**Figure 2.**
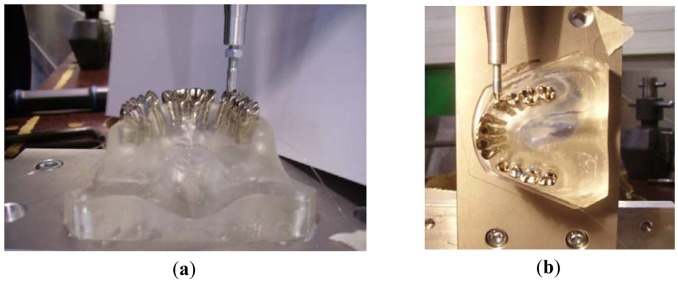
Photograph of the maxilla's model during the tests with the Universal Test Machine over the canine: (**a**) vertically applied force; (**b**) laterally applied force.

Before measuring the forces transmitted by the orthodontic appliances to the teeth and bone, the sensors response were verified by using a Universal Test Machine. Well known controlled forces were applied of the teeth's crown at the vertical direction (Figure 2(a)) coinciding with the longest axis of the root and also laterally to the teeth (Figure 2(b)). The range of forces applied in the vertical direction ranged between 0 and 100 N and in the lateral direction from 0 to 200 N. The second step included tests using the dentition instrumented by an orthodontic appliance, including brackets, arch wire and an extra-oral anchorage. The system of loading here is promoted by the activation of the arch wire (tie of the wire and opening of coils) or by applying an extra-oral appliance (head gear).

The gratings were interrogated in reflection using a broad band optical source and an optical spectral analyzer [8]. Forces were estimated by correlating the wavelength shifts as function of the applied force. To determine the peak positions of the spectrum and increase the measurement reliability, a specially designed radial-basis function network was used [14].

## 3. Orthodontic and Orthopedic Forces

**Figure 3.**
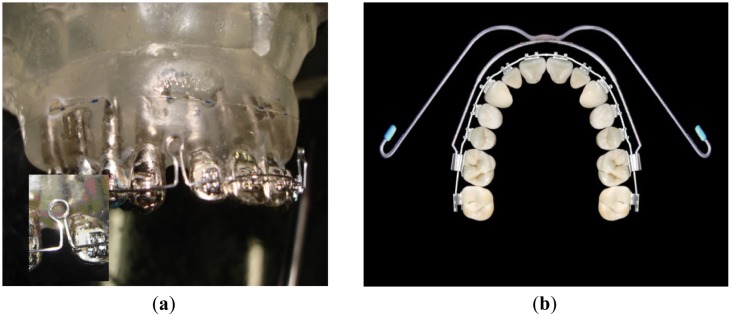
(**a**) Picture of the maxilla model instrumented with brackets an arch wire (squared cross section) and two loop-coils; Inset: the zoom-in picture of a loop-coil opened by 3 mm for activation; (**b**) Occlusal image of the teeth with fixed appliance and extra-oral device connected at the first molars.

To observe how the forces are transmitted to the teeth and bone when the orthodontic system is activated the maxilla model was instrumented with orthodontic appliances. The appliance used was an Edgewise Standard bracket (Figure 3(a)) and with an arch wire (squared cross-section with 0.019 × 0.026 inches sides) designed to have two coils (loop) at both sides (Figure 3(a) inset). The arch wire is designed to close the space left by the premolars teeth extraction, which was done to provide space to align the incisors and canines. In a real treatment, the coils are opened 3 mm when the arch is adapted to the brackets and tied in the back face of the posterior teeth. The closing of the coils makes the anterior teeth to move backwards to the posterior direction closing the spaces left by the extracted teeth. As a consequence of the arch wire activation, it applies pressure to the teeth, promoting movement and bone remodeling. In the artificial model there isn't bone remodeling or even teeth displacement. Hence the wavelength variation (Δλ) measured by the gratings corresponds to the initial forces resulting from the installation and activation of the appliances. The experiments evaluated the change of the FBG's Bragg wavelength as function of the arch wire activation (coils opening). To extend the test a real treatment was simulated using extra-oral forces with a standard head gear through appliance's anchorage outside of the mouth (Figure 3(b)).

After processing the gratings spectral data, characteristics associated to very small deformation on the order of 4 με can be observed which could further be converted into mechanical forces. To determine the range of the forces it was applied the equation *F/A* = *E*ε. Where the *F* is the applied force, *A* is the area, *E is* the elasticity modulus and ε is the fiber relative deformation.

The measurements with the orthodontic appliance were repeated three times for each tooth: incisor, canine and molar, all of them at the left side of the maxilla model. The loading procedure followed the mechanical test changing only the range of the forces from 0 to 2,600 gf (25.49 N).

## 4. Experimental Results

**Figure 4.**
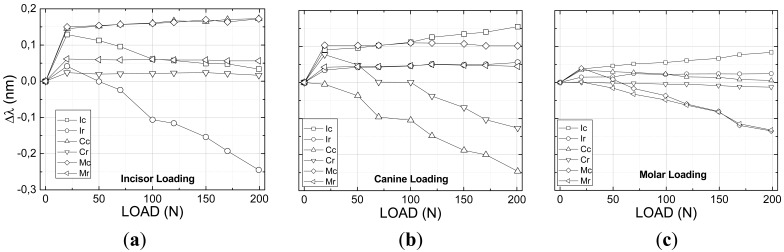
Change of the Bragg wavelength for each of the gratings as a function of the vertical load applied to the different teeth: (**a**) incisor; (**b**) canine; (**c**) molar.

**Figure 5.**
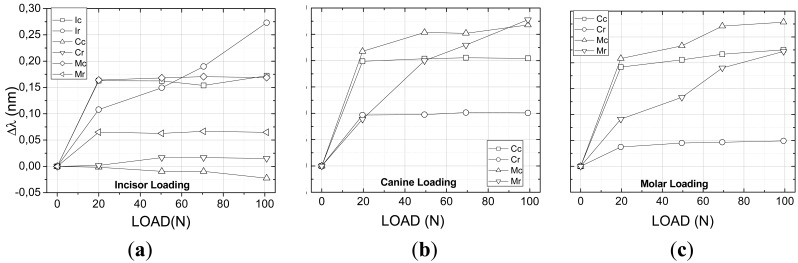
Change of the Bragg wavelength for each one of the gratings as a function of the lateral load applied to different teeth: (**a**) incisor; (**b**) canine; (**c**) molar.

From the mechanical tests using the Universal Test Machine it was possible to determine the maxilla model response as a result of the applied forces at the two directions, vertical (Figure 4(a–c)) and lateral (Figure 5(a–c)).

Figure 4 shows the results of all six sensors placed at the teeth, both at the crown and apex, for vertical loading of the incisor, canine and molar teeth. As expected all teeth that are directly receiving the applied loading sense compressive forces both at the root, near to the crown, and at the apex. It is observed however that for small loads either the apex or the root might have an inverse behavior which can be account for as a result of the system conformation. As the loading increases both regions are solely subjected to compressive forces. The other teeth which are not directly subjected to the applied force experience some level of traction as a result of the polymer material pulling the teeth surrounding the compressed region. The maximum wavelength shift was measured to be on the order of 0.25 nm. The smallest forces were measured for the molar loading where the sensors at the canine tooth were subjected to negligible forces.

The multiplexed fiber sensors FBG a, b, c and d did not experience any deformation which means that probably the tensions were not transferred to the surface of the material representing the maxillary material bone. Table 2 summarizes the final wavelength shift experienced by the gratings.

The lateral tests were conducted also on the incisor, canine and molar teeth following the same protocol as for the first longitudinal tests using a range of forces between 0 to 10 N (Figure 5(a)). As a result of the lateral forces all sensors measured traction forces independently on which tooth received the applied force. The molar tooth showed the highest deformation for both the canine and molar loading especially at the apex region where the sensors presented wavelength shifts on the order of 0.27 nm (Figure 5(b,c)) probably attributed to the tension transmitted across the elastomeric material representing the alveolar bone forcing the root of the molar. The apex region of the incisor also presented a high level of traction when the load is applied directly over it. As a general feature the canine tooth presented the lowest deformation with some compression for the incisor loading (Figure 5(a)). It is noted that the forces applied at the molar crown affected the canine tooth pretty much the same way that the direct loading did and the highest response is found near to the crown. No deformation was observed at the incisor tooth for the lateral loading of the canine and molar teeth and therefore the data points were removed of the graphs for clarity. As observed for the vertical loading the gratings (a, b, c and d) inside of the model monitoring what is called the alveolar bone did not experience any mechanical force during the mechanical lateral loading tests.

In both cases of mechanical loading the maxilla model a difference is normally observed between forces measured in the apex and near to the crown of the teeth resulting in a force gradient along the tooth length which might also contribute to the bone formation during an orthodontic treatment.

### 4.1. Real Orthodontic Forces—The Application of an Orthodontic Appliance

**Figure 6.**
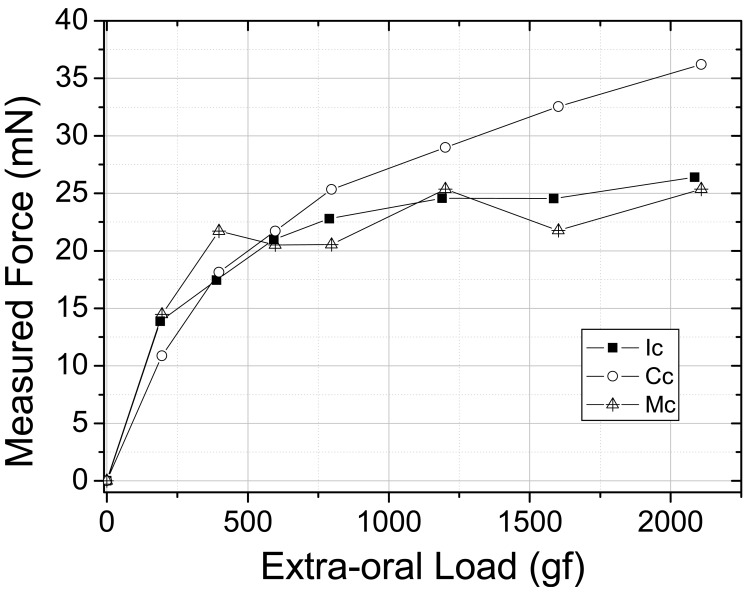
Forces at the roots of incisor, canine and molar teeth as function of the arch wire activation and extra-oral applied loading (orthodontic and orthopedic appliances).

In a second approach, the intention was to monitor internal forces within the maxilla model when real external orthodontic forces are applied. The forces from the orthodontic appliance were obtained by the activation of the arch wire by tying the arch wire up at its extremity at the first molars and consequently opening the loop-coils by 3 mm and promoting tension from the arch to the teeth (Figure 3(a)). This activation is responsible for the first tension rise shown in Figure 6 when the tension measured by the sensors goes from 0 to 10–15 mN. Afterwards the extra-oral appliance is coupled to the fixed appliance and loaded from approximately 240 to 2,100 g and the sensors responses were monitored. The graph in Figure 6 shows the magnitude of the force as a function of the orthodontic and orthopedic activation (gf). All three teeth were sensible to the extra-oral appliance loading and the maximum forces produced by the fixed extra-oral appliances were measured to be of approximately 25 mN for the incisor and molar teeth and 35 mN for the canine tooth. In this case the extra-oral appliance was subjected to 2,100 g of load which is a condition found in a standard orthodontic treatment.

From the orthodontic forces (fixed appliance) and orthopedic forces (extra-oral appliance) it was possible to observe response on teeth roots between 0 and 35 mN. On the other hand no tensions from orthodontics and orthopedics forces were detected at the surface of the maxilla model by the FGB a, b, c and d sensors (Table 4).

## 5. Conclusions

During a real orthodontic and orthopedic treatment the bone remodeling and tooth movement are force level dependent and there are some hypothesis that associates side affects like hearing organ pain or tooth absorption to the level of forces. The results of this laboratory study suggest that the forces levels observed are in accordance with levels found in the literature to induce a tooth movement. Also the events correlated to the bone remodeling should occur based on isolated mechanisms and not only local factors, as local tensions. It is important to distinguish local tensions and the use of orthopedics appliances as they can be responsible for tissue injures or pain.

As it was expected the FBGs sensors monitored tensions at the teeth roots from external orthodontics and orthopedics forces as well as the FBG sensors at the maxillas' surface were not affected by the orthodontics and orthopedic forces. This study provide a new way to observe external forces dissipated in internal artificial structures of the teeth and bone and it is especially important for giving arguments to correlate orthopedic forces (extra-oral appliance) and side effects. Even more relevant information concerning variables that affect clinical practice in dentistry, prostheses and orthodontic questions can benefit from using optical fiber sensors.

## Figures and Tables

**Table 1.** Positioning and monitoring wavelength of the gratings instrumented at the maxilla model.

**Position**	**Tooth Crown**	**Tooth Root**
**Incisor**	Ic_FBG_ (1,531 nm)	Ir_FBG_ (1,543 nm)
**Canine**	Cc_FBG_ (1,520 nm)	Cr_FBG_ (1,534 nm)
**Molar**	Mc_FBG_ (1,540 nm)	Mr_FBG_ (1,554 nm)
**Alveolar Bone**	FBG a, b, c, d.	Transversally

**Table 2.** Mechanical vertical loading tests.

**Loading**	**Tooth Root**	**Final Δλ**	**Apex**	**Final Δλ**
**Incisor**	Ic	0.03 nm	Ir	−0.25 nm
**Canine**	Cc	−0.20 nm	Cr	−0.25 nm
**Molar**	Mc	−0.14 nm	Mr	−0.14 nm
**Alveolar Bone**	FBG a, b, c, d—There was no wavelength displacement.			

**Table 3.** Mechanical lateral loading testes.

**Loading**	**Tooth Root**	**Final Δλ**	**Apex**	**Final Δλ**
**Incisor**	Ic	0.15 nm	Ir	0.25 nm
**Canine**	Cc	0.20 nm	Cr	0.10 nm
**Molar**	Mc	0.25 nm	Mr	0.20 nm
**Alveolar Bone**	FBG a, b, c, d—There was no wavelength displacement			

**Table 4.** Orthodontic testes.

**Loading**	**Tooth Root**	**Force: 2,100 g**
**Incisor**	Ic	25 mN
**Canine**	Cc	35 mN
**Molar**	Mc	25 mN
**Alveolar Bone**	FBG a, b, c, d—There was no wavelength displacement	
